# The hyperornithinemia–hyperammonemia-homocitrullinuria syndrome

**DOI:** 10.1186/s13023-015-0242-9

**Published:** 2015-03-11

**Authors:** Diego Martinelli, Daria Diodato, Emanuela Ponzi, Magnus Monné, Sara Boenzi, Enrico Bertini, Giuseppe Fiermonte, Carlo Dionisi-Vici

**Affiliations:** Division of Metabolism, Children Research Hospital Bambino Gesù, Rome, Italy; Neuromuscular and Neurodegenerative Diseases Unit, Children Research Hospital Bambino Gesù, Rome, Italy; Medical Genetics Division, Catholic University Policlinico Gemelli, Rome, Italy; Sciences Department, Università della Basilicata, Potenza, Italy; Biosciences, Biotechnologies and Biopharmaceuticals Department, Biochemistry e Molecular Biology Lab, Università di Bari, Bari, Italy

**Keywords:** HHH syndrome, Urea cycle disorders, Hyperammonemia, *SLC25A15*, ORC1, ORNT1

## Abstract

**Background:**

Hyperornithinemia-hyperammonemia-homocitrullinuria (HHH) syndrome is a rare autosomal recessive disorder of the urea cycle. HHH has a panethnic distribution, with a major prevalence in Canada, Italy and Japan. Acute clinical signs include intermittent episodes of vomiting, confusion or coma and hepatitis-like attacks. Alternatively, patients show a chronic course with aversion for protein rich foods, developmental delay/intellectual disability, myoclonic seizures, ataxia and pyramidal dysfunction. HHH syndrome is caused by impaired ornithine transport across the inner mitochondrial membrane due to mutations in *SLC25A15* gene, which encodes for the mitochondrial ornithine carrier ORC1. The diagnosis relies on clinical signs and the peculiar metabolic triad of hyperammonemia, hyperornithinemia, and urinary excretion of homocitrulline. HHH syndrome enters in the differential diagnosis with other inherited or acquired conditions presenting with hyperammonemia.

**Methods:**

A systematic review of publications reporting patients with HHH syndrome was performed.

**Results:**

We retrospectively evaluated the clinical, biochemical and genetic profile of 111 HHH syndrome patients, 109 reported in 61 published articles, and two unpublished cases. Lethargy and coma are frequent at disease onset, whereas pyramidal dysfunction and cognitive/behavioural abnormalities represent the most common clinical features in late-onset cases or during the disease course. Two common mutations, F188del and R179* account respectively for about 30% and 15% of patients with the HHH syndrome. Interestingly, the majority of mutations are located in residues that have side chains protruding into the internal pore of ORC1, suggesting their possible interference with substrate translocation. Acute and chronic management consists in the control of hyperammonemia with protein-restricted diet supplemented with citrulline/arginine and ammonia scavengers. Prognosis of HHH syndrome is variable, ranging from a severe course with disabling manifestations to milder variants compatible with an almost normal life.

**Conclusions:**

This paper provides detailed information on the clinical, metabolic and genetic profiles of all HHH syndrome patients published to date. The clinical phenotype is extremely variable and its severity does not correlate with the genotype or with recorded ammonium/ornithine plasma levels. Early intervention allows almost normal life span but the prognosis is variable, suggesting the need for a better understanding of the still unsolved pathophysiology of the disease.

**Electronic supplementary material:**

The online version of this article (doi:10.1186/s13023-015-0242-9) contains supplementary material, which is available to authorized users.

## Background

Hyperornithinemia-hyperammonemia-homocitrullinuria (HHH, MIM #238970) syndrome is a rare genetic disorder of the urea cycle (UC) caused by mutations in the *SLC25A15* or *ORNT1* gene (MIM*603861), which encodes for the mitochondrial ornithine carrier ORC1 [1]. HHH syndrome represents a heterogeneous disease with high clinical variability, ranging from a mild form with learning difficulties and slight neurological involvement, to a more severe form with coma, lethargy, hepatic signs and seizures. Asides from the severe neonatal form, there is no evidence of a direct correlation between age of onset, which is variable, and disease severity [1]. As for other urea cycle disorders (UCDs), early diagnosis in infancy or childhood may improve the clinical outcome [1]. Acute treatment requires an emergency approach, whereas the long-term treatment consists of a low-protein diet supplemented with citrulline or arginine; in some patients, sodium benzoate and/or sodium phenylbutyrate are used to maintain blood ammonia in a safe range [1]. In this paper, we aim to provide a comprehensive review of the genetic and molecular aspects of HHH syndrome, a descriptive picture of clinical features and therapeutic strategies along with a discussion on the still unsolved questions related to the disease pathomechanisms.

### History and disease definition

In 1969, Shih et al. described a 3 years-old boy with cognitive impairment and myoclonic seizures, in whom intermittent hyperammonemia was associated with abnormal high plasma ornithine levels and homocitrullinuria [2]. These authors coined the name “hyperornithinemia-hyperammonemia-homocitrullinuria (HHH) syndrome” (Orpha number 415) to describe the peculiar biochemical profile, suggestive of a “block in the ornithine metabolic pathway”.

### Epidemiology

Since the original description [2-4], more than 100 patients with HHH syndrome have been reported [5-61]. Overall, according to a recent survey based on newborn screening data on over 6 million births in United States (US) and data from two large US and European longitudinal registries, the incidence of all UCDs is estimated as 1:35.000 live births [62]; however figures reporting the incidence of HHH syndrome are still lacking. Based on the few available large series studies on UCDs, HHH syndrome accounts for 1% – 3,8% of all UCDs [62,63]. On the basis of the available information in the literature for 97 out of 111 patients [5-61] the male/female ratio is approximately 2:1 (Table 1).

**Table 1:** **The table summarizes relevant clinical features and molecular findings in 111 patients with HHH syndrome**

**Pt**	**Sex**	**Ethnicity**	**Onset years**	**Diagnosis years**	**Vital status** **(last age reported)**	**Lethargy**	**Coma**	⇑ **ASAT** ⇑ **ALAT**	**Coagulopathy**	**Intellectual disability**	**Seizures,** **myoclonic**	**Pyramidal signs**	**Plasma ornithine at diagnosis**	**Mutation effect at protein level**	**Ref.**
1	m	English/French Canadian	1,1	1,5	43,0	Yes	No	Yes	No	1	Yes	Yes	915	p.A264P/p.F188del	[2-4]
2	f	English	2,5	21,0	23,0	Yes	Yes	No	No	3	Yes	Yes	1021		[5,6]
3	m	French Canadian	0,8	7,0	9,0	Yes	Yes	Yes	Yes	3	Yes	Yes	372		[7-12]
4	m	French Canadian	1,9	2,0	5,0	Yes	Yes			3	Yes	Yes	330		[7,9,11]
5	f	French Canadian		47	17,0	Yes	No			1	No	No	380		[7,11]
6	m	French Canadian		31,0*	31,0	No	No			3	Yes		519		[7]
7	f	French Canadian		22,0*	22,0	yes	No			No	No	No	430		[7]
8	m	French Canadian		18,0*	19,0	No	No		Yes+	3	No	No	483		[7]
9	f	NA		4,0	6,5	No	No	Yes	No	No	No	No	410		[13]
10	f	Italian	0,2	0,8	1,0	Yes		No	No	1	Yes	Yes	689		[14]
11	m	NA	1,4	2,5	4,7	Yes	No	No		3	Yes	No	229		[9]
12	m	Italian	1,1	2,5	Death 2,5	Yes	Yes	Yes+	Yes+			Yes	797		[15]
13	m	Italian	0,8	2,6	3,6	Yes		No	No	No		Yes	1041		[15]
14	m	Japanese		42,0	46,0	Yes	Yes	No					700		[16]
15	m	NA		5,5	8,0	No	No	No	No	3	Yes	Yes	586		[17]
16	m	NA								No			1014		[18]
17	m	NA		4,2	10,7					1					[18]
18	m	Norwegian/Yugosl-Italian	0,4	1,1	4,0	No	No	Yes+	Yes	No	No	No	887		[19]
19	f	Italian	Birth	0,2	20,0	Yes	Yes	Yes	Yes	No	No	Yes	618	p.G27R/p.G27R	[20-23]
20	m	Japanese		13,0*	13,0	No	No	No	No	1	Yes	Yes	409		[24]
21	m	Japanese	4,0	19,0	19,0	Yes	No	No	No	3	Yes	Yes	477		[24]
22	m	Spanish	9,0	21,0	21,0		No	No		3	No	Yes	719		[25]
23	f	Spanish		18,0	18,0		No	No		3	No	Yes	780		[25]
24	m	Spanish	4,0	10,0	13,0	Yes	Yes	No	NA	3		Yes	713		[25]
25	m	Italian	Birth	2,0	21,0	Yes	Yes	Yes	No	3	Yes	Yes		p.Ser90*/p.Ser90*	[21-23]
26	m	Japanese	3,0	10,0	10,0	Yes	No	No	No	3	No	Yes	419	p.R179*/p.R179*	[26,27]
27	f	Japanese			37,0								504		[28]
28	m	Japanese			11,0								879		[28]
29	m	Japanese			19,0								553		[28]
30	f			20,0	24,0						Yes	Yes	860		[29]
31	m	Japanese		17,0	17,0	Yes	No			3	Yes	Yes	834	p.229_230insN/ p.229_230insN	[30,31]
32	m			37,0	39,0	Yes	Yes	Yes	Yes	No	No	Yes	310		[32]
33	f			40,0*	42,0	Yes	No			No			263		[32]
34	m	Japanese		35,0	41,0	Yes	Yes	No		1	No	Yes	586	p.R179*/p.R179*	[31,33]
35	m	Japanese		15,0	16,0		No	No		3	No	Yes		p.G27E/p.G27E	[31,34]
36	m	French Canadian	Birth	Birth	Death Birth	Yes	Yes	Yes					1915		[35]
37	m	French Canadian		1,2	21,0	No	No	Yes	Yes	1	No	Yes	727	p.F188del/p.F188del	[36-38]
38	f	French Canadian		5,3	22,0	No	No	Yes	Yes	1	No	Yes	343	p.F188del	[36-38]
39	f	French Canadian		1,3	25,0	No	No	Yes	Yes	1	No	Yes	1083	p.F188del/p.F188del	[36-38]
40	m	French Canadian		12,0	33,0	No	No	No	No	3	No	Yes	515	p.F188del/p.F188del	[36-38]
41	m	French Canadian		3,5	35,0	No	No	Yes+	No	3	No	Yes	606	p.F188del/p.F188del	[36-38]
42	m	French Canadian		8,0	40,0	Yes	Yes	Yes	Yes+	3	Yes	Yes	700	p.F188del/p.F188del	[36-39]
43	f	French Canadian	3,5	5,5	6,5	No	No	Yes	Yes	1	No	Yes	470		[40]
44		NA	0,6	0,6						3			577		[41]
45	m	Italian	Birth	Birth	10,0	Yes	Yes	No	No	No	Yes	Yes	595	p.G27R/p.Tyr55	[22,23,42]
46		Japanese/ Irish												p.E180K/large del	[37]
47		Palestinian	Birth	Birth		Yes	Yes							p.A15E/p.A15E	[38,43]
48	f	South American												p.M33QfsX1/p.M33QfsX1	[38,43]
49	m	Italian	12,0	26,0	28,0	Yes	No	No	Yes	1	Yes	Yes		p.R179*/p.R179*	[22,23]
50	m	Italian	18,0	21,0	29,0	Yes	Yes	Yes+	No	1	Yes	Yes	505	(IVS5 + 1 g > a/IVS5 + 1 g > a) exon skipping	[22,23]
51	f	Italian	3,0	7,0	24,0	Yes	Yes	No	No	2	Yes	Yes	780	p.R275Q/p.R275Q	[22,23,44]
52	f	Italian	1,0	33,0	44,0					3		Yes		p.Q89*/p.Q89*	[22,23]
53	m	Italian	Birth	Birth	23,0	Yes	Yes			No	Yes	Yes		p.F188del/p.G190D	[22,23]
54	f	NA	5,0	17,0	32,5		No	No	No	3	No	Yes	312		[45]
55	m	NA	13,0	15,0	15,0		No	No		3	No		520		[45]
56	f	NA		0,8	0,8		No	No	No	1	No	No			[46]
57	f	Japanese		52,0	52,0	No	No	No	No	No	Yes	Yes	373	p.R179*/p.R179*	[27,47]
58	m	Slovakian		1,6	10,0	No	No	Yes+		No	No		425	p.G113C	[48]
59	m	Japanese	1,5	15,0	15,0		No	No	No	3	No	Yes	682	p.P126R/p.P126R	[49]
60	m	Palestinian		1,8	1,8		No	Yes	No	3	Yes	No	532	p.L193*/p.L193*	[50]
61	m	Palestinian		13,0*	13,0		No	No	No	3	Yes	No	314	p.L193*/p.L193*	[50]
62	f	Japanese	2,0	30,0	30,0		No			2	Yes	Yes	373	p.R275*/p.R275*	[51]
63	m	Japanese	3,0	34,0	Death 34,0	Yes	Yes			3	Yes	Yes		p.R275*/p.R275*	[51]
64	m	Italian	3,5	3,5	3,5	Yes	No	Yes+	Yes+	No	No	No	852	p.G113C/p.M273K	[52]
65	m	Mexican		5,0*	9,0		No	Yes+	Yes	No	No	No	697	p.T32R/p.T32R	[53]
66	f	Mexican		13,0	18,0		No			No	No	No	353	p.T32R/p.T32R	[53]
67	m	Mexican		8,0	Death 21,0	Yes	Yes			3	No	Yes	386	p.T32R/p.T32R	[53]
68	m	Mexican		7,0*	20,0		No			No	No	No	371	p.T32R/p.T32R	[53]
69	m	Mexican		3,0*	15,0		No			No	No	No	370	p.T32R/p.T32R	[53]
70	m	NA	Birth	0,1	3,0		Yes			3			616		[54]
71	f	French-Canadian	1,0	1,1	1,1	Yes	No	Yes+	Yes+	No	No	No	933	p.F188del/p.F188del	[55]
72	f	French-Canadian	1,2	1,2	1,2	No	No	Yes+	Yes+	No	No	No	580	p.F188del/p.F188del	[55]
73	f	Saudi arabia	1,0	1,5	4,0	No	No	Yes+	Yes+	3	No	Yes	465	p.G220R/p.G220R	[56]
74		Saudi arabia		13,0*	13,0	No	No			1	No	Yes	590	p.G220R/p.G220R	[56]
75		Saudi arabia		7.0*	7,0	No	No			1	No	Yes	493	p.G220R/p.G220R	[56]
76		French Canadian		1,0	14,0	No	No	Yes+	Yes+		No	Yes	642	p.F188del/p.F188del	[38]
77		French Canadian		3,6	13,0	No	No	Yes+	Yes		No	Yes	432	p.F188del/p.F188del	[38]
78		French Canadian		2,4	9,0	No	No	Yes+	Yes+		No	Yes	310	p.F188del/p.F188del	[38]
79		French Canadian		0,2**	7,0	No	No	No	No		No	Yes	397	p.F188del/p.F188del	[38]
80		French Canadian		2,0	6,0	No	No	Yes	No		No	Yes	337	p.F188del/p.F188del	[38]
81		French Canadian		15,0	29,0	Yes	Yes	No	Yes		Yes	Yes	431	p.F188del/p.F188del	[38]
82		French Canadian		16,0	Death 23,0	Yes	Yes	Yes			No	Yes	227	p.F188del/p.F188del	[38]
83		French Canadian		2,0	31,0	No	No				No	Yes	581	p.F188del/p.F188del	[38]
84		French Canadian		3,0	17,0	No	No				No	Yes	529	p.F188del/p.F188del	[38]
85		French Canadian		1,5	5,0	No	No	Yes			No	Yes	348	p.F188del/p.F188del	[38]
86	m	Belgium	0,3	0,7	6,0	No	No	Yes	Yes	1	No	No	951	p.M37R/p.M37R	[57]
87	f	Italian	Birth	0,2	Death 0,2		Yes	No	No	No	No	No		p.L71Q/p.L71Q	[57]
88	m	Algerian		2,0	5,0		No	No	No	3	No	Yes	885	p.K245*/p.K245*	[57]
89	m	Algerian	Birth	Birth*	3,0		No	No	No	3	No	Yes	887	p.K245*/p.K245*	[57]
90	f	Senegal	Birth	Birth	6,0	Yes	Yes	No	Yes+	1	Yes	Yes	509	p.R179*/p.R179*	[57]
91	f	Senegal	Birth	Birth	6,0	Yes	No	No	No	1	No	Yes	290	p.R179*/p.R179*	[57]
92	m	Spanish		2,0	2,0					3			419	p.G216S/p.G216S	[57]
93	f	Taiwan	1,1	2,0	2,0	Yes	Yes	Yes+	Yes+				450	p.T272I/p.T272I	[57]
94	m	Italian		41,0	41,0	Yes	Yes	No	Yes	No	No	Yes	216	p.G27R/p.G27R	[57]
95	m	Italian	24,0	54,0	54,0	Yes	No	Yes+	Yes	No	No	Yes	603	(c.56 + 1G > T/intronic change) exon skipping	[57]
96	f	USA-Greece	Birth	Birth	0,1	Yes	Yes	No	Yes	No			370	p.S175fsX192/p.L283F	[57]
97	m	Morocco		1,2	1,5		Yes	Yes+	Yes+	1	No	Yes	700	p.A70L/p.A70L	[57]
98	f	Pakistan		1,0	5,0	No	No	Yes	No	3	No	No	471	p.F188L/p.F188L	[57]
99	m	Morocco	1,7	1,7	2,0	Yes	No	Yes+	Yes	No	No	No	493	p.R179*/p.R179*	[57]
100	m	Morocco		4,0*	4,0									p.R179*/p.R179*	[57]
101	f	Pakistan	20,0	57,0	57,0	Yes	Yes	No	No	No	Yes	Yes		p.A70L/p.A70L	[57]
102	m	Indian	35,0	35,0	35,0	Yes		No	No	No		No	292	p.G220R/p.R275*	[58]
103	f	French Canadian	2,0	15,0	57,0	Yes	Yes	Yes			Yes	Yes	542	p.F188del/p.A264P	[4]
104	f	El Salvador	4,4	4,5	Death 31,0	Yes	No	Yes+	Yes	2	Yes	Yes	521	p.M33Qfs*1/M33Qfs*1	[4]
105	f	Vietnam	1,0	5,0	29,0	Yes	Yes	Yes	Yes	2	Yes	Yes	1439	p.R179*/p.R179*	[4]
106	m	Italian	6,0	36,0	36,0	Yes						Yes	309	p.F188del/p.L193P	[59]
107	m	Turkish	3,5	6,0	9,0	Yes	No	No	No		No		380	p.A15V/p.A15V	[60]
108	m	Han Chinese	1,0	2,0	6,0	No	No	Yes	Yes	No	No	No	503	p.R179*/p.T272I	[61]
109	m	Han Chinese		Birth*	3,0	No	No	No	No	No	No	No		p.R179*/p.T272I	[61]
110	f	Italian	1,0	8,0	8,0	No	No	Yes	No	1	No	Yes	427	p.G27R/p.R275G	Unp.
111	f	Italian	1,0	4,0*	4,0	No	No	No	No	No	No	Yes	531	p.G27R/p.R275G	Unp.

Yes + means severe abnormality; age at diagnosis includes *prospective diagnosis and **newborn screening; intellectual disability (score: 1, mild; 2, moderate; 3, severe); Unp. means unpublished; ⇑ means increased.

## Methods

We retrieved clinical and investigational data on HHH syndrome patients published to date. The PubMed database was searched using the terms: “HHH syndrome; Hyperornithinemia; Hyperammonemia; Homocitrullinuria; ornithine carrier; ORC1; *ORNT1*; urea cycle disorders; *SLC25A15*”. The terms were variably combined with: “early onset, infancy-childhood onset; adult onset, late onset, review, case, case series”. In addition, references listed in the papers retrieved by this method as well as in the textbook “The Metabolic and Molecular Bases of Inherited Diseases” [64] were screened, along with a reference list started in 1985 by the last author of this work. Data on clinical symptoms and biochemical and genetic data were pooled for analysis whenever the description of the cases allowed. The individual relevant data obtained from the literature were used to create a database that is reported in Table 1 and in Additional file 1: Table S1. We additionally included data from two unreported patients, recently diagnosed and managed at Children’s Research Hospital Bambino Gesù, Rome, Italy (patients 110 and 111); written informed consent was obtained from the caregivers of these two patients for publication of their clinical and biochemical data. Patients n. 10, 19, 25, 49, 51, 52, and 94 were monitored longitudinally in the same institution.

## Results

### Ethnic distribution

Although the disease has a panethnic distribution, it has been more frequently reported in three countries: 25 patients (23%) were Canadian, as a result of a founder mutation in Quebec [37], 18 patients (17%) were Italian and 14 patients (13%) were Japanese. Therefore, these three countries account for more of 50% of affected cases. The complete list of patients’ ethnic background is displayed in Table 1.

### Clinical description

Age at onset, type and severity of the symptoms in HHH syndrome are highly variable. Clinical symptoms usually start from early infancy, including the neonatal period, to childhood and, more rarely, in adulthood.

### Age at onset and diagnosis

**Figure 1:**
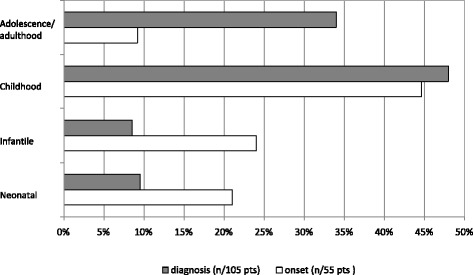
**The graph shows the age at onset** **(white bars)** **and the age at diagnosis** **(gray bars)** **of HHH syndrome patients.** Patients are divided into four age categories: neonatal (birth —1 month) infantile (> 1 month—1 year), childhood (> 1 years— 12 yrs), and adolescence/adulthood (> 12 years). Values are expressed as percentage of the total.

The retrospective review of the literature provided information on the age at onset in 54 HHH syndrome patients and on the age at diagnosis in 105 subjects (Table 1), whom we arbitrarily divided into four categories: neonatal (birth – 1 month), infantile (>1 month – 1 year), childhood (>1 years – 12 years), and adolescence/adulthood (>12 years). In 14 patients there was a prospective diagnosis, because of an affected sibling or previous familial HHH syndrome cases; only one patient was identified by newborn screening. Figure 1 shows the percentage of patients grouped into the four categories at onset and at diagnosis, respectively. As shown in the figure, 22% had a neonatal presentation, 24% infantile, 44% manifested the disorder in childhood, and 9% in adolescence/adulthood. Although symptoms began most frequently in neonatal age/infancy (46% of patients), the diagnosis was often delayed with at least one fourth of cases identified in adulthood. Remarkably, in one third of patients with neonatal onset of symptoms, the diagnosis was delayed into subsequent diagnostic age categories. By comparing the age at onset of the clinical symptoms with the age of diagnosis for those cases in which both these data were available, there was a mean diagnostic delay of 6.3 ± 10.1 years (range 0 – 37 years).

### Acute presentation

**Figure 2:**
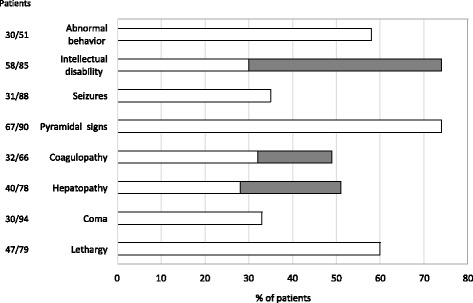
**The graph shows the frequency of clinical features in HHH syndrome.** For Intellectual disability we identified two categories: mild (lQ/DQ50-69)/modarate (IQ/DQ35-49) [white bars], and serve (IQ/DQ < 35) [gray barsj. A similar classification was adopted for hepatopathy [mild/moderate up to 10×) or serve (>10×) increase of trans saminases] and coagulopathy [mild:nioderate individua coagulation factors 40%-70%, INP 1.5-2.0) or serve (individuaI coagulation factors <40%, INP > 2.0 or related clinical Manifestations) abnormalities of prothromibin time and INR).

As seen in other UCDs [1], in the acute phase the HHH syndrome combines hyperammonemia with tachypnoea, respiratory alkalosis, feeding and gastrointestinal problems, ataxia, lethargy, confusion, and coma. About 1/3 of patients experienced an overt episode of coma and many others had recurrent episodes of lethargy (Figure 2). Coma and lethargy at onset are quite common in the earlier onset group (about 70%), becoming progressively less frequent in patients with later onset. Variable neurological symptoms may characterize the acute presentation and include seizures, dysphasia, movement and gait disturbances, drop-attacks and behavioural changes [38,57,58].

As shown in Figure 2, HHH syndrome may also exhibit as fulminant liver failure with severe coagulation abnormalities (e.g. subdural hematoma, gingival bleeding, melena) and/or as hepatitis-like attacks [15,23,25,35,37,38,50,52,57]. Remarkably, in some cases massive elevation of transaminases, with or without signs of acute liver failure (i.e. coagulation abnormalities with prolonged prothombin time), occurred in the absence of overt hyperammonemia [15,38,53,55,57].

### Chronic presentation

HHH syndrome may also present a more chronic and slowly progressive course, characterized by an aversion to protein-rich foods, progressive encephalopathy with mental regression and signs of motor dysfunction (Figure 2). Affected patients in the late-onset categories come to medical attention mainly for evaluation of intellectual disability (ID), recurrent vomiting or neurological findings like ataxia and seizures.

### Neurological complications

The peculiar feature of HHH syndrome is a progressive neurological dysfunction characterized by pyramidal tract signs with spastic gait, associated with cerebellar symptoms and myoclonic seizures [23,36,38]. Regardless of the age and type of onset, pyramidal dysfunction has been reported in about 2/3 of patients (Figure 2). This feature can vary from lower limb hyperreflexia with positive Babinski sign, with or without gait abnormalities, to a clear picture of spastic paraparesis. Cerebellar signs include ataxia, poor hand and fine motor coordination, dysdiadochokinesia, dysarthria, nystagmus and intentional tremor [4,23,36,38]. Other neurological signs include muscle weakness, involuntary hand movements, buccofaciolingual dyspraxia and psychiatric disorders. Seizures, mainly myoclonic, are present in 35% of patients (Figure 2) and seem to be more frequent in those with an earlier onset of the disease [4,14,51,59].

Cognitive impairment is also often reported in this disorder, and may range from major developmental delay to variable degree of ID [23,36,38]. Independently from the age of onset, a greater proportion of patients (74%) presented with cognitive defect, being mild/moderate in 36% and severe in 38% of patients, respectively (Figure 2). A normal intellectual development was recorded in 29 out of 86 patients (34%). However, some patients with normal cognitive development displayed behavioral problems [15,32]. The degree of ID doesn’t seem to be proportionally related either to the frequency of lethargy/coma episodes or to ammonium or ornithine concentrations in plasma, as suggested in literature [38]. Additional clinical features more rarely reported in some HHH syndrome patients include dysmorphic features, cerebral dysplasia [5,6,46] and microcephaly [14,57].

### Ocular findings

Retinal involvement, with photophobia, hemeralopia, tapetoretinal degeneration, cataract and abnormal electroretinogram has also been reported [37,38,44].

### Aetiology

#### Biochemical derangement and pathophysiology

**Figure 3:**
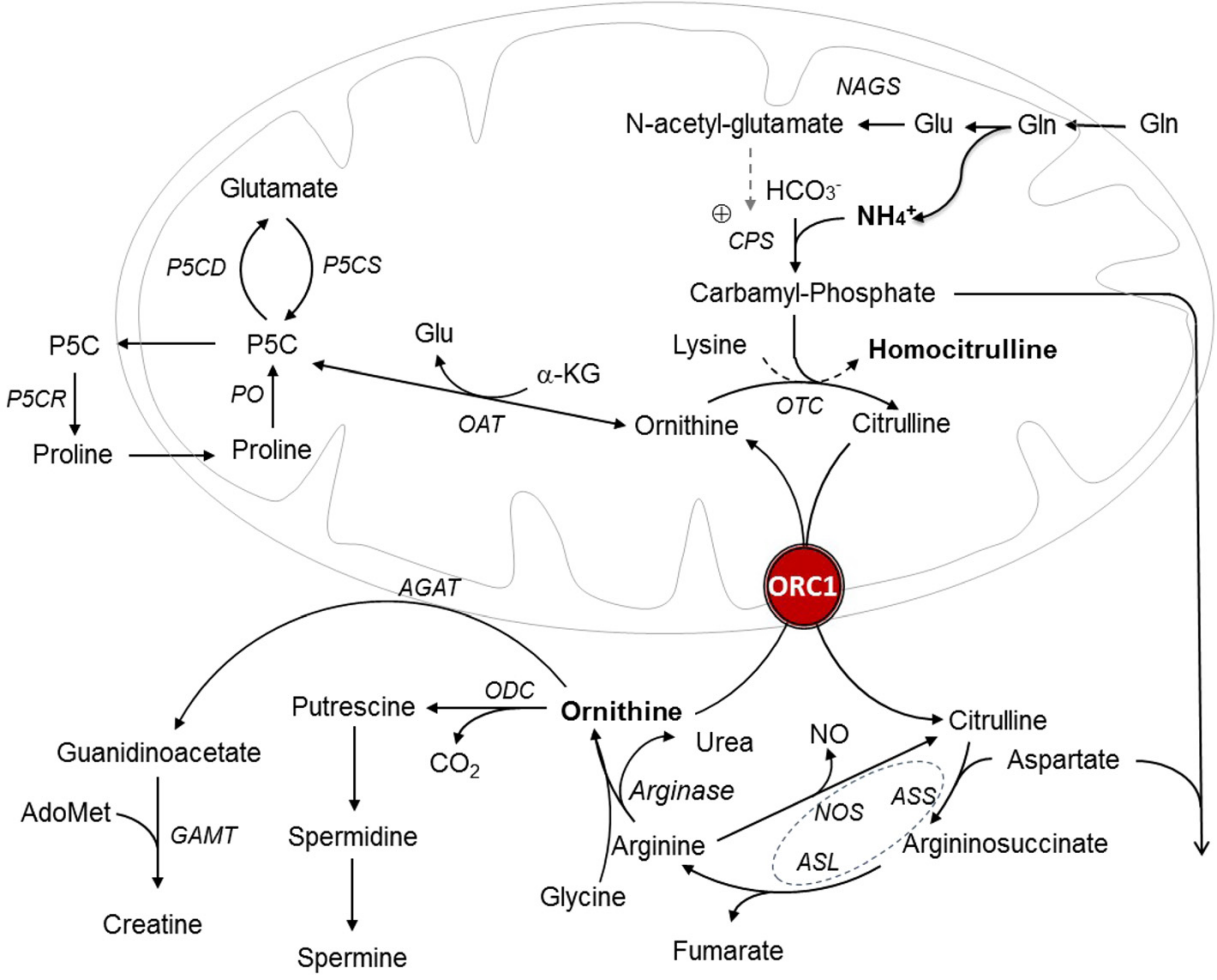
**The urea cycle and related patwhays.** The acronyms correspond to: NAGS, N-acetylglutamate synthase; CPS, carbamyl-phosphate synthetase; OTC omithine transcarbamylase; ORC1, ornithine carrier 1; ASS argininosuccinate synthetase; ASL argininosuccinate lyase; NOS, nitric oxide synthase: ODC ortnithine decarboxylase; AGAT, argine: glycine amidinotransferase; GAMT, guanidinoacetate N-methyltransferase; OAT, ornithine delta-aminotransferase; P5CD 1- pyrrolin-e5-carboxylate dehydrogenase; P5CS, 1-pyrroline-5 carboxylate synthetase; P5CR,1-pyrroline-5-carboxylate reductase; PO, proline oxidase. The dashed circle indicates the multiprotien complex, which also includes cationic aminoacid transporter 1 (CAT1) and heat shock protein 90 (HSP 90).

The biochemical role of ORC1 is complex and highly relevant for the different tissues where it is expressed. ORC1 transports ornithine, lysine and arginine into the mitochondrial matrix of peripheral tissues and pericentral hepatocytes; in periportal hepatocytes, in which UC enzymes are expressed, it catalyzes a very efficient ornithine/citrulline exchange reaction [65], connecting the enzyme activities of urea synthesis in the cytosol to those in the mitochondria. ORC1 plays therefore a key role in the UC (Figure 3). ORC1 catalyzes the transport of the L-isomers of ornithine, citrulline, lysine and arginine by a 1:1 substrate exchange reaction and less efficiently exchanges a basic amino acid for an H+ [65-67]. Two human isoforms of the mitochondrial ornithine carrier, ORC1 and ORC2, have been identified so far. Despite having a high sequence identity (87%) with ORC1, ORC2 is less active, presents a lower affinity for ornithine and citrulline, and shows a broader substrate specificity because of its capability to transport histidine and homocitrulline as well as the D-isomers of ornithine, lysine and arginine [68]. Both isoforms are mainly expressed in liver, pancreas, lungs, and testis, although ORC2 to a much lesser extent than ORC1 in all tissues investigated [68]. The total mitochondrial ornithine/citrulline exchange activity per whole organ *in vivo* is unknown; it has been suggested that the late onset and the variable clinical phenotype of HHH syndrome may be due to the redundancy of this exchange activity [8,37]. This is catalyzed either by ORC2 [68] or by the *SLC25A29* gene product (previously reported to be a mitochondrial carnitine/acylcarnitine- or ornithine-like carrier called ORNT3 [69]), which is able to rescue the ornithine metabolism deficiency in fibroblasts of HHH patients [69,70] and to transport basic amino acids as well as ornithine into proteoliposomes [71]. The residual ornithine transport in cultured fibroblasts and liver of affected individuals supports this hypothesis of gene redundancy in HHH syndrome [8,37].

ORC1 deficiency reduces the mitochondrial availability of ornithine, which increases in the cytosol causing hyperornithinemia. The accumulation of ornithine in cytosol leads also to increased levels of polyamines as spermine and spermidine [72]. In the periportal zone of the liver lobuli, the lower level of mitochondrial ornithine reduces the UC rate since the matrix ornithine is required by the ornithine transcarbamylase (OTC) to prime the UC. Ammonia and carbamyl-phosphate (CP) levels increase, explaining hyperammonemia. Increased CP may have two destinations: it may bind lysine, forming homocitrulline which gives rise to homocitrullinuria or enter in the pyrimidine pathway, leading to increased excretion of orotic acid in urine (Figure 3). Plasma glutamine is taken up from the systemic circulation into the gut and then processed to glutamate, pyrroline-5-carboxylate, ornithine and citrulline. As the liver extracts only a small quantity of citrulline, this aminoacid is mainly transported to the kidney and metabolized to arginine in the tubules of the cortical zone and used for guanidino acetate synthesis by arginine-glycine amidotransferase (AGAT), the first step of creatine synthesis, with ornithine as by-product [73]. In ORC1 deficiency, the high cytosolic ornithine concentrations inhibit AGAT leading to secondary creatine deficiency [21,44,74]. Secondary creatine deficiency may be also due to low cellular arginine availability due to the block in UC flow. Moreover, ornithine excess may inhibit creatine biosynthesis as observed in ornithine delta-aminotransferase (OAT) deficiency [75,76] (Figure 3). This is in line with a previous observation of two patients with HHH syndrome, in whom urinary creatine excretion was normalized by citrulline and arginine but not by ornithine supplementation [21].

Since ORC1 is highly expressed in the brain and in particular in astrocytes, one could speculate that an 7absent or dysfunctional protein may affects the metabolism of neurons and of glial cells. Hyperammonemia has a toxic effect on the astrocytes, causing mitochondrial dysfunction, cellular swelling and a change in cellular bioenergetics [77]. Moreover, Braissant et al. [78] have shown through in vitro experiments with reaggregated mixed brain cells (neurons, astrocytes, oligodendrocytes and microglia) primary cultures, impaired axonal growth and abnormal localization and phosphorylation of the intermediate neurofilament M-protein after ammonia exposure. Addition of creatine to the media seems to protect against this effect of ammonia on the neural cytoskeleton; this protective effect depends on the presence of glial cells and cannot be observed in neuron enriched cell cultures (78). These findings suggest that in ORC1 deficiency AGAT inhibition due to high ornithine levels reduces the endogenous creatine production in cerebrum and cerebellum and thus renders the brain more vulnerable to a local increase of ammonia [78].

Recently, Viegas et al. have shown that excessive ornithine and homocitrulline levels can cause protein and lipid oxidation and may negatively interfere in oxidative phosphorylation and Krebs cycle function of the rat brain, with secondary oxidative stress [79,80]. Impairment of brain bioenergetics and the oxidative damage induced by these metabolites may possibly contribute to the neurological symptoms affecting patients with HHH syndrome.

#### Molecular genetics

**Figure 4:**
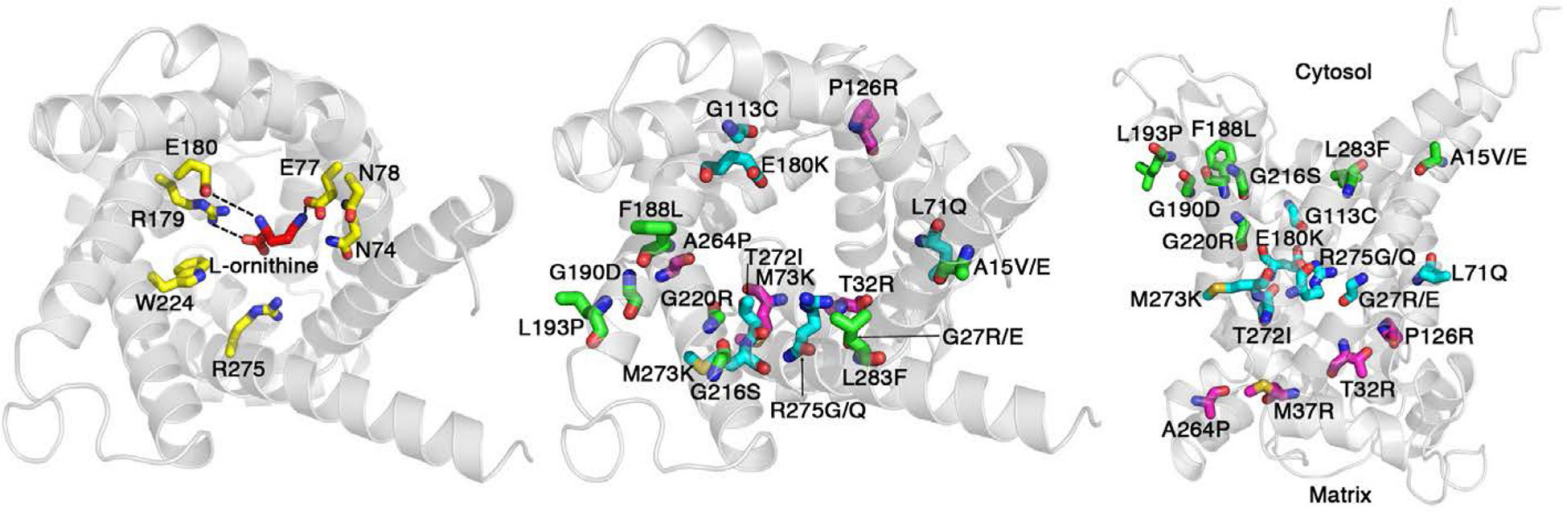
**Human ORC1 homology model.** Left panel shows the ORCI model, created as dcflbed in [80], viewedfroni the cytoplasnijc side ith the substrate-binding site containing L- ornithine. The central panel shows the location of the mutations causing the H H H syndrome in the ORC1 model viewed as in the left panel but displayed with residues close to the binding site colored in cyan, residues close to the substrate exit/entrance on the cytosolic side in green and residues close to the substrate exit/entrance on the matrix side in magenta. The right panel shows the ORC1 model. viewed from the membrane side with the color scheme as in the central panel.

HHH syndrome is a genetic autosomal recessive disease caused by mutations in the *SLC25A15* (solute carrier family 25, member 15) gene [37]. This gene maps on chromosome 13q14.11, spans about 23 kb and contains 7 exons encoding for the isoform 1 of the ornithine carrier ORC1, a member of the mitochondrial carrier family [65]. The open reading frame is encoded by exons 2 through 7 [53]. Exon 1 encodes part of the 5’UTR. The normal product of *SLC25A15* gene is a 301 amino acid protein composed, like other mitochondrial carriers, of six α-helices that traverse the inner mitochondrial membrane with the C- and N-termini exposed to the cytosolic side of the membrane. ORC1 shows a tripartite structure with three similar domains each with two transmembrane helices connected by a long hydrophilic matrix loop [65]. There is experimental evidence for the direct involvement of the ORC1 residues E77, R179 and E180 in substrate binding, whereas W224 and R275 seem to be important in triggering the substrate-induced conformational changes that leads to substrate translocation; N74 and N78 residues are part of the substrate binding pocket [81] (Figure 4).

Several *SLC25A15* mutations have been associated with the clinical phenotype of the HHH syndrome. From 1999 to 2014, 35 mutations have been identified: 18 missense mutations, 7 small insertion, 2 small deletions, 4 nonsense mutations, 1 gross deletion, 1 micro-rearrangement, 1 intronic rearrangement (Table 1). All mutations occurred in the coding region. Two common mutations, p.F188del and p.R179*, are reported. The first accounts for about 30% of patients with the HHH syndrome and it is characteristic (but not exclusive) of patients of French-Canadian descent because of a founder effect [38]. The second mutation (15% of HHH patients) appears to be prevalent in patients of Japanese and Middle Eastern origin [27,47,57].

The functional effects of some *SLC25A15* mutations on substrate transport have been investigated using *in vitro* cell culture and liposome reconstitution studies [53,57,68], revealing that some *SLC25A15* missense and nonsense mutations (p.T32R, p.F188del, p.G190D, p.M273K, p.T272I, p.G113C, p.L71Q and p.L283F) cause reduced transport activity while others (p.G220R, p.R179*, p.G27R, p.R275Q and R275*) completely abolish the function. Interestingly, some patients with null *SLC25A15* alleles did not show neonatal hyperammonemia [57].

The 18 pathogenic missense mutations alter residues phylogenetically conserved in the wild-type protein, which may reflect that they have specific important roles for function. However, the majority of the single-residue mutations causing the HHH syndrome are non-conservative substitutions that in many cases introduce a change in the charge of amino acid side chain (p.A15E, p.G27R, p.G27E, p.T32R, p.M37R, p.P126R, p.E180K, p.G190D, p.G220R, p.M273K, p.R275G and p.R275Q) [22,37,57], prolines (p.L193P and p.A264P) or residues with different side chain size or polarity (p.A15V, p.G113C, p.G216S, p.T272I, p.L71Q, p.F188L and p.L283F) [57,60], that alter the protein structure and function. Missense mutations can be mapped onto the 3D structural homology model of ORC1 [81], which is based on the structure of the carboxyatractyloside inhibited bovine ADP/ATP carrier (PDB ID 1OKC), to display the location of each mutation (Figure 4). In a simple analysis, the mutations may be divided into three groups depending on their location in the model: residues close to the proposed binding site, residues close to the substrate entrance/exit on the cytosolic side and residues close to the substrate exit/entrance on the matrix side. The majority of the HHH syndrome mutations are found in residues that have side chains protruding into the internal pore of ORC1 where the substrate is translocated, suggesting that these mutations could interfere with substrate translocation. Mutations located in residues of the substrate binding site p.E180K, p.R275G and p.R275Q are presumably directly deleterious to substrate binding and other mutations in proximity, such as p.G27R, p.G27E, p.T272I and p.G113C, probably have an effect in deforming the substrate-binding pocket. Mutations on the cytosolic side, such as p.F188L, p.G190D, p.G216S and p.G220R, could interfere with the substrate entering or exiting its binding site, or with the closing/opening of the substrate-binding cavity towards the cytosol. A similar explanation could be given for the effect of p.T32R and p.A264P mutations that interfere with the closing/opening of the substrate-binding cavity but on the matrix side. The residues A15, M37, L71, P126, L193 and M273 have side chains on the external surface of ORC1 and therefore these mutations cannot directly obstruct substrate translocation. Instead these mutations may indirectly impair function, probably by causing local perturbations in the ORC1 structure, e.g., the proline of the p.P126R mutation is located in one of the signature motifs that are highly conserved in all mitochondrial carriers and is likely to be crucial for the structure and conformational changes during substrate translocation [72].

### Diagnosis

#### Biochemical profile

The metabolic triad of hyperammonemia, hyperornithinemia, and urinary excretion of homocitrulline establishes the diagnosis of HHH syndrome. However, some patients may present with an incomplete biochemical phenotype. The biochemical data in individuals with HHH syndrome at the time of diagnosis are summarized in Table 2 and in Additional file 1: Table S1. Of note, HHH syndrome is characterized by a lower degree of hyperammonemia if compared with other UCDs [1] and plasma ammonia level usually normalizes in response to pharmacological treatment.

**Table 2:** **Biochemical data** (**mean** ± **SD**, **range**, **and number of patients**) **detected in plasma of HHH syndrome patients at diagnosis**

**Metabolites**	**HHH patients**	**Reference value**
**Ammonia**	215 ± 279 (18–2300) n = 93	<55
**Ornithine**	575 ± 265 (216–1915) n = 96	30-110
**Citrulline**	26 ± 14 (1–57) n = 21	10-52
**Glutamine**	1149 ± 592 (447–3688) n = 43	333-809
**Arginine**	66 ± 26 (27–126) n = 22	20-112
**Lysine**	178 ± 177 (35–858) n = 26	80-257

Values are expressed as μmol/L.

Plasma concentration of ornithine can range from 216 to 1915 μmol/L (normal: 30–110 μmol/L). Despite protein-restricted diet in combination with pharmacological treatment, ornithine concentration in plasma remains elevated with only a very few patients reported with normal levels at long-term follow-up [2-4,15,48,56].

Homocitrullinuria is a hallmark of the disease, however some patients may show absent or only minimal excretion of homocitrulline in urine [50] (Additional file 1: Table S1).

Similarly to other UCDs [1], plasma glutamine concentrations and urinary orotic acid may be elevated (Additional file 1: Table S1). Occasional organic aciduria, with increase excretion of Krebs Cycle intermediates (succinate, citrate, fumaric, α-ketoglutaric) and lactate was reported [50,52].

#### Study of intracellular mitochondrial transport of radiolabelled ^14^C-ornithine in cultured skin fibroblasts

The diagnosis of HHH syndrome may be confirmed by the evaluation of cellular mitochondrial transport of radiolabelled ^14^C-ornithine in cultured skin fibroblasts [9]. Cultured fibroblasts from patients with null mutations usually show an approximately 75%-80% reduction in ornithine transport: this may suggest a potential role of redundant transporters in achieving a residual transport [37,69]. There is no correlation between ornithine transport capacity, genotype and phenotype [37,53].

#### Other investigations

As detected by computer tomography (CT) scan and magnetic resonance (MRI) studies, brain abnormalities include mild cerebral and cerebellar atrophy [16,23,31,27,57], white matter changes [4,57,59], subdural hematoma [C. Dionisi-Vici, personal observation], cystic lesions and calcifications [4], and diffuse brain edema, evident at ultrasound scan [35]. However, normal brain CT scan or MRI findings were reported in several patients, mainly in those who did not experience an overt hyperammonemic coma [36,40,60]. Interestingly, multiple stroke-like lesions were reported in a 4 year-old patient presenting irritability, vomiting, highly elevated liver enzymes, hyperammonemia, and coagulopathy [56].

Neurophysiologic studies show abnormal motor evoked potentials affecting pyramidal tract of lower limbs, generally without upper limb involvement [24,36]. Somatosensory evoked potentials are normal; electroneuromyography can show abnormal nerve conduction velocity [24,36,37,51] and may reveal signs of spinal anterior horn cells degeneration [34].

Blood coagulation studies may be abnormal with deficiency of factor VII, X, XI, and antithrombin III [7,15,21,38,39,55-57].

Liver structural (light microscopy) and ultrastructural changes (electron microscopy) are commonly observed [7,9,26,40,57,82] and include vacuolated hepatocytes with intracytoplasmic glycogen deposition, small nuclei, dense chromatin, and fat droplets without fibrosis. At electron microscopy, mitochondria appear abnormally shaped and sized with lamellar cristal-like inclusions [11].

### Differential diagnosis

#### Hyperammonemia

HHH syndrome enters in the differential diagnosis of any patient presenting with hyperammonemia at any age. The main causes of hyperammonemia associated with inborn errors of metabolism include [1,83]: UCDs (they generally present along with elevation in plasma ammonia concentration, hyperglutaminemia and metabolic alkalosis); organic acidemias (they show in addition metabolic acidosis, ketonuria and normal or reduced plasma glutamine levels); fatty-acid oxidation defects (hyperammonemia is associated with hypoglycaemia, hypertransaminasemia, increase of plasma creatine-phosphokinase levels); lysinuric protein intolerance (characterized by low concentrations of plasma ornithine, lysine, and arginine and persistent urinary excretion of lysine, ornithine, and arginine); hyperinsulinism-hyperammonemia syndrome (characterized by severe hypoglycaemia); pyruvate carboxylase deficiency (presenting with lactic acidosis and hypoglycaemia); and the recently reported defect of mitochondrial carbonic anhydrase VA (presenting with lactic acidosis, hypoglycaemia and a characteristic organic aciduria) [84].

A complete routine chemistry panel [i.e. blood ammonia, glucose, lactate, liver function tests (including transaminases, gamma-GT, coagulation, bilirubin, albumin) creatine kinase, uric acid, arterial blood gases, and blood cell counts, urinalysis], and metabolic investigations with plasma amino acids, urine amino acids,organic acids and orotic acid measurements allow the differential diagnosis.

#### Hyperornithinemia

Hyperornithinemia is also a characteristic feature of OAT deficiency responsible for gyrate atrophy of the choroid and retina (OMIM #258870) [85]. This disorder is caused by defect in *OAT* gene, located at 10q26. The fasting plasma ornithine in OAT deficiency ranges from 400 to 1400 μmol/L [85]. Initial symptoms include myopia and night blindness, starting in early to mid-childhood; patients then develop reduction of visual fields, posterior subcapsular cataracts and abnormal electroretinogram [85]. The fundoscopic aspect of the chorioretinal atrophy in gyrate atrophy is highly specific. Patients have generally a normal cognitive level, although one large series suggests an increased incidence of intellectual impairment [76]. A few OAT patients experienced neonatal hyperammonemia [86]. Interestingly, and similarly to some HHH patients, they were all found to have normal plasma ornithine concentration at birth which progressively increased in the following weeks when the diagnosis was fully defined [86].

#### Homocitrullinuria

Other conditions in which homocitrullinuria can be observed should be listed in the differential diagnosis of HHH syndrome. These include lysinuric protein intolerance (MIM# 222700) or OTC deficiency (MIM# 311250), which can present in some cases with low quantity of homocitrulline in urine [87,88]. Furthermore, canned food or heated milk products may represent a source of detectable homocitrulline in urine [88].

#### Neurologic findings

In patients with early childhood onset of gait abnormalities and spasticity, the differential diagnosis includes cerebral palsies and inherited spastic paraplegias. Interestingly among the inherited disorders of the UC, HHH syndrome and argininemia share the peculiar neurological feature of pyramidal dysfunction [1,88,89].

### Newborn screening

In some countries, like United States and Canada, HHH syndrome is included in the disease panel of expanded newborn screening programs. However, it may be missed on newborn screening because some affected neonates may not show in the first days of life elevated plasma ornithine levels that can be detected by tandem mass spectroscopy [90]. Authors suggested that the typical rise of plasma ornithine levels occurs after the first few days of life when blood samples for newborn screening are obtained.

### Genetic diagnosis

Genetic testing is the gold standard to confirm the diagnosis. The *SLC25A15* gene was cloned in 1999 and a common F188del mutation in French Canadian patients was identified [37]. Genetic analysis does not have a prognostic value since even in the same family and with the same mutation the phenotype can be quite variable. Sequence analysis is performed first, followed by deletion/duplication analysis if only one or no mutant *SLC25A15* alleles are detected. The gold standard for prenatal diagnosis in couples of known genotype is mutation analysis. Before the discovery of the molecular basis of HHH syndrome, prenatal diagnosis has been performed by the analysis of ornithine incorporation in cultured amniotic fluid cells [35,41].

### Management

*Acute treatment* is similar to other UCDs [1]. Protein intake must be stopped for 24 h and intravenous glucose in combination with first-line medications must be started. Arginine (and/or citrulline) must be administered to replace the missing UC intermediates and to allow protein synthesis. Ammonia scavengers as sodium benzoate and sodium phenylbutyrateare used for bypassing the UC block. *Long*-*term treatment* is based on a low-protein diet supplemented with citrulline or arginine; ornithine supplementation has been tried in the past with contradictory results [4,18,21,24,25,31] in the attempt to correct ornithine depletion in the mitochondria, however its use is not recommended. Protein restriction may be combined with sodium benzoate or sodium phenylbutyrate. If plasma creatine levels are low, creatine supplementation should be instituted [1,44,74]. Citrulline supplementation has been shown to allow better metabolic control and to avoid secondary creatine deficiency [21]. The retrospective review of the literature provided information on treatment modalities in HHH patients. All were treated with low protein diet, 22% with protein restriction alone. Thirty per cent of patients received arginine supplementation, 22% citrulline and 10% ornithine; 36% were treated with ammonia scavengers, (21% benzoate, 12% phenylbutyrate, and 3% combined benzoate and phenylbutyrate). To our knowledge, liver transplantation was done only in one HHH patient with severe metabolic derangement [54]. The main parameters to be monitored at follow-up are similar to other UCDs [1], and include plasma ammonia, plasma aminoacids and urinary orotic acid.

### Prognosis

Prognosis is highly variable ranging from mild neurological involvement to a severely disabling disease (Table 1). Out of the 106 patients with a known vital status (median age 14 years, range 1 month – 57 years), 7 patients died, representing an overall mortality rate of the 6,6%. An appropriate management with protein restriction diet, supplements and ammonia scavengers allows almost normal life duration. Treated patients are usually metabolically stable and do not experience relapses of hyperammonemia [38]. Hepatic signs resolve rapidly with treatment and long-term hepatic function is normal [52]. Chronic therapy prevents hyperammonemia and liver disease but does not affect the spastic paraparesis [21,22,38]. Successful pregnancies have been reported in HHH syndrome female patients [4,7,29].

### Unresolved questions

Although the disease responds well to treatment with low risk of relapse of hyperammonemia [38], slowly progressive pyramidal signs characterize the chronic course, as also seen in argininemia [89]. However, the mechanism(s) of pyramidal dysfunction in HHH syndrome still remains to be elucidated. Creatine deficiency may contribute to the pathogenetic mechanism of the syndrome, as creatine is relevant for mitochondrial energy metabolism, regulation of glycolysis, proteins synthesis, membrane stabilization and neuromodulation [77,78,85]. This could be in line with the finding of abnormally shaped mitochondria at electron microscopy studies in skin fibroblasts, hepatocytes and muscle biopsy from HHH syndrome patients [11,23,82]. Furthermore, a mitochondrial dysfunction has been recently related to the effects of ornithine and homocitrulline in causing oxidative stress and disturbed mitochondrial homeostasis [79,80].

A further mechanism that can be involved in the pathophysiology of HHH syndrome is related to polyamines metabolism. Shimizu and colleagues reported increased total and fractional (putrescine, cadaverine, spermine, spermidine) polyamines in one HHH syndrome patient [30]. Indeed, the clinical similarities between HHH syndrome and argininemia, which has been associated to an abnormal polyamine metabolism [91,92], may suggest a common pathogenetic mechanism causing pyramidal dysfunction.

Overall, the pathogenesis of HHH syndrome is complex and not completely understood. It is likely that different mechanisms, including the impact of low mitochondrial ornithine on UC flux, the presence of hyperammonemic crises and the disturbance of other pathways in major organs play a role in determining the heterogeneous clinical presentation of ORC1 deficiency.

In addition, as molecular studies failed to disclose a correlation between type of mutations or ornithine transport capacity and disease severity, an effect of genetic modifiers, such as *ORC* genes redundancy, seems to be likely, but further studies are certainly needed to clarify this point.

Interestingly, there is a report describing hyperammonemic encephalopathy in two consanguineous weanling foals presenting postweaning with anorexia, abnormal behaviour, unthriftiness, and poor growth [93]. Hyperammonemia was associated with increased transaminases, abnormal coagulation, and mild hyperbilirubinemia. The animal presented with severe neurologic abnormalities, including circling, bilateral forelimb and hindlimb ataxia, and dementia. Biochemical investigations revealed an aminoacid profile consistent with HHH syndrome with elevated serum ornithine and glutamine, homocitrullinuria and orotic aciduria, highlighting the strong similarities between the human disease and this spontaneous animal model.

## Conclusions

In this article, we have retrospectively reviewed the clinical and metabolic profiles of all reported HHH syndrome patients. The combination of peculiar clinical features and the biochemical triad of hyperammonemia, persistent hyperornithinemia, and urinary excretion of homocitrulline, allows the diagnosis of HHH syndrome. Encephalopathy, coagulopathy and liver disease are common acute features, whereas pyramidal dysfunction and cognitive disabilities typically characterize the chronic course of the disease. The clinical phenotype is extremely variable and its severity does not correlate with the genotype or with recorded ammonium/ornhitine plasma levels. The pathophysiological mechanisms underlying HHH syndrome have not been fully elucidated yet, but they seem to include features common to both primary UCDs as well as to mitochondrial disorders.

## Additional file

Additional file 1: Table S1. The table summarizes the relevant laboratory findings at diagnosis in 111 patients with HHH syndrome. Values of ammonia and glutamine are expressed as μmol/L. Urinary orotate and homocitrulline (when possible) are expressed as μmol/mmol creatinine.

## Abbreviations

AGAT: Arginine-glycine amidotransferase
CP: Carbamyl-phosphate
HHH: Hyperornithinemia- hyperammonemia-homocitrullinuria
ID: Intellectual disability
OAT: Ornithine delta-aminotransferase
ORC1: Ornithine carrier 1
OTC: Ornithine transcarbamylase
UC: Urea cycle
UCDs: Urea cycle disorders
