# Supplementary material for: The hyperornithinemia–hyperammonemia-homocitrullinuria syndrome
Source: Orphanet J Rare Dis. 2015 Mar 11;10:29. doi: 10.1186/s13023-015-0242-9 (PMC4358699; doi:10.1186/s13023-015-0242-9)
Supplement: Additional file 1: Table S1. — The table summarizes the relevant laboratory findings at diagnosis in 111 patients with HHH syndrome. Values of ammonia and glutamine are expressed as μmol/L. Urinary orotate and homocitrulline (when possible) are expressed as μmol/mmol creatinine. [file 13023_2015_242_MOESM1_ESM.docx]

| **Patient** | **Blood ammonia** | **Plasma glutamine** | **Urine homocitrulline** | **Urine Orotate** | **Ref.** |
| --- | --- | --- | --- | --- | --- |
| 1 | 88 | 340-558 | 307 μm/day |  | 2-4 |
| 2 | 141 |  | 1058 μm/day |  | 5-6 |
| 3 | 99- 279 | 850 | 32 |  | 7-12 |
| 4 | 148 | 1032 | 83 |  | 7,9,11 |
| 5 | 99 - 158 | 962 | 24 |  | 7,11 |
| 6 | 119 | 844 | 31 |  | 7 |
| 7 | 111 | 803 | 134 |  | 7 |
| 8 | 80 | 931 | 56 |  | 7 |
| 9 | 150 |  |  |  | 13 |
| 10 | 70 |  |  |  | 14 |
| 11 | 96 | 711 |  |  | 9 |
| 12 | 152 |  |  |  | 15 |
| 13 | 87 - 151 | 1130 | 280 μm/day |  | 15 |
| 14 | 88 - 217 |  |  |  | 16 |
| 15 | 88 |  |  |  | 17 |
| 16 | 68 - 176 |  |  | 255 - 1500 | 18 |
| 17 |  |  |  |  | 18 |
| 18 | 156 | 741 | 269 |  | 19 |
| 19 | 29 |  | Detectable | 37 | 20-23 |
| 20 | 48 - 190 | not increased | 48 | 168 | 24 |
| 21 | 105 - 294 | not increased | 78 | 103 | 24 |
| 22 | 26 - 164 | 1216-1653 | 410-598 μm/day | 8 - 12 | 25 |
| 23 | 36 - 136 | 1340-1409 | 1053-2484 μm/day | 20 - 47 | 25 |
| 24 | 763 | 608-1243 | 427-566 μm/day | 7 - 32 | 25 |
| 25 | 240 |  |  |  | 21-23 |
| 26 | 109 - 200 |  |  |  | 26-27 |
| 27 |  |  | 63 | 12 | 28 |
| 28 |  |  | 42 |  | 28 |
| 29 |  |  | 48 |  | 28 |
| 30 | >300 |  | 643 μm/day |  | 29 |
| 31 | 100 - 350 | 370-513 | Detectable |  | 30,31 |
| 32 | 318 | 1370 |  |  | 32 |
| 33 | 25 | 1650 |  |  | 32 |
| 34 | 98 |  | 4.57 μm/L |  | 31,33 |
| 35 | 1108 |  |  |  | 31,34 |
| 36 | 1107 | 3688 |  | 342 | 35 |
| 37 | 109 | 852 | Detectable |  | 36-38 |
| 38 | 119 | 900 | Detectable |  | 36-38 |
| 39 | 217 | 850 | Detectable |  | 36-38 |
| 40 | 64 | 1303 | Detectable |  | 36-38 |
| 41 | 325 | 1145 | Detectable |  | 36-38 |
| 42 | 216 | 1249 | 117 |  | 36-39 |
| 43 | 108 - 206 | 809 | 83 | 723 | 40 |
| 44 | 101 |  |  | 39 | 41 |
| 45 | 317 | 981 | 317 | 1209 | 22,23,42 |
| 46 |  |  |  |  | 37 |
| 47 |  |  |  |  | 38,43 |
| 48 |  |  |  |  | 38,43 |
| 49 |  |  |  |  | 22,23 |
| 50 | 509 |  |  |  | 22,23 |
| 51 |  |  |  | 15 | 22,23,44 |
| 52 |  |  |  |  | 22,23 |
| 53 |  |  |  |  | 22,23 |
| 54 | 50 | 1489 | 903 μm/L |  | 45 |
| 55 | 60 |  | 35 |  | 45 |
| 56 |  |  |  |  | 46 |
| 57 | 74 - 237 |  |  |  | 47 |
| 58 | 71 - 80 |  |  | 14 - 295 | 48 |
| 59 | 70 - 117 |  |  |  | 49 |
| 60 | 194 | 447 | 208 | 334 | 50 |
| 61 | 18 | 735 | 220 | 3 | 50 |
| 62 | 82 |  |  |  | 51 |
| 63 |  |  |  |  | 51 |
| 64 | 171 |  | 34 | 43 | 52 |
| 65 | 100 | 1255 |  | 25 | 53 |
| 66 | 40 | 1184 |  | 1 | 53 |
| 67 | 55 | 1573 |  | 3 | 53 |
| 68 | 43 | 1071 |  | 14 | 53 |
| 69 | 45 | 1038 |  | 4 | 53 |
| 70 | 2300 | 1355 |  |  | 54 |
| 71 | 53 - 139 |  | 456 μm/L |  | 55 |
| 72 | 98 |  |  |  | 55 |
| 73 | 532 | 1101 | 108 | 1210 | 56 |
| 74 | 77 | 765 | 13 | 3 | 56 |
| 75 | 54 | 630 | 21 | 20 | 56 |
| 76 | 49 |  |  |  | 38 |
| 77 | 139 |  |  |  | 38 |
| 78 | 54 |  |  |  | 38 |
| 79 | 173 |  |  |  | 38 |
| 80 | 315 |  |  |  | 38 |
| 81 | 125 |  |  |  | 38 |
| 82 | 250 |  |  |  | 38 |
| 83 | 100 |  |  |  | 38 |
| 84 | 120 |  |  |  | 38 |
| 85 | 58 |  |  |  | 38 |
| 86 | 137 |  | Detectable |  | 57 |
| 87 |  |  |  |  | 57 |
| 88 | 370 |  |  | 4 | 57 |
| 89 | 125 |  |  | 207 | 57 |
| 90 | 700 |  | Detectable | 109 | 57 |
| 91 | 180 |  | Detectable | 1 | 57 |
| 92 | 75 |  | Detectable | 12 | 57 |
| 93 | 337 |  | Detectable | 5 | 57 |
| 94 | 235 |  |  |  | 57 |
| 95 | 222 |  | Detectable | 12 | 57 |
| 96 | 400 |  |  | 78 | 57 |
| 97 | 200 |  | Detectable |  | 57 |
| 98 | 62 |  | Detectable | 69 | 57 |
| 99 | 96 |  | Detectable | 75 | 57 |
| 100 |  |  |  |  | 57 |
| 101 | 306 |  |  |  | 57 |
| 102 | 140 | 1322 |  | Absent | 58 |
| 103 | 346 | 679-1177 | 208-302 |  | 4 |
| 104 | 521 | 359-824 | 58-250 |  | 4 |
| 105 | 588 | 667-3169 | 177 |  | 4 |
| 106 |  |  |  |  | 59 |
| 107 | 300 |  | 385 |  | 60 |
| 108 | 132 |  | 71 |  | 61 |
| 109 | 68 |  |  |  | 61 |
| 110 | 65 | 1137 |  | Normal | Unp. |
| 111 | 22 | 973 |  | 34 | Unp. |

**Supplementary Table 1**. The table summarizes the relevant laboratory findings at diagnosis in 111 patients with HHH syndrome. Values of ammonia and glutamine are expressed as µmol/L. Urinary orotate and homocirulline (when possible) are expressed as µmol/mmol creatinine.
